# The novel hyaluronic acid granular hydrogel attenuates osteoarthritis progression by inhibiting the TLR‐2/NF‐κB signaling pathway through suppressing cellular senescence

**DOI:** 10.1002/btm2.10475

**Published:** 2022-12-23

**Authors:** Chen Zhang, Zhengxiang Cheng, Yuanyuan Zhou, Ziyi Yu, Hongyu Mai, Changhao Xu, Jing Zhang, Jiali Wang

**Affiliations:** ^1^ School of Biomedical Engineering, Sun Yat‐sen University Guangzhou People's Republic of China; ^2^ State Key Laboratory of Materials‐Oriented Chemical Engineering College of Chemical Engineering, Nanjing Tech University Nanjing People's Republic of China

**Keywords:** cellular senescence, granular hydrogel, hyaluronic acid, osteoarthritis, TLR‐2

## Abstract

In patients with mild osteoarthritis (OA), two to four monthly injections are required for 6 months due to the degradation of hyaluronic acid (HA) by peroxidative cleavage and hyaluronidase. However, frequent injections may lead to local infection and also cause inconvenience to patients during the COVID‐19 pandemic. Herein, we developed a novel HA granular hydrogel (n‐HA) with improved degradation resistance. The chemical structure, injectable capability, morphology, rheological properties, biodegradability, and cytocompatibility of the n‐HA were investigated. In addition, the effects of the n‐HA on the senescence‐associated inflammatory responses were studied via flow cytometry, cytochemical staining, Real time quantitative polymerase chain reaction (RT‐qPCR), and western blot analysis. Importantly, the treatment outcome of the n‐HA with one single injection relative to the commercial HA product with four consecutive injections within one treatment course in an OA mouse model underwent anterior cruciate ligament transection (ACLT) was systematically evaluated. Our developed n‐HA exhibited a perfect unification of high crosslink density, good injectability, excellent resistance to enzymatic hydrolysis, satisfactory biocompatibility, and anti‐inflammatory responses through a series of in vitro studies. Compared to the commercial HA product with four consecutive injections, a single injection of n‐HA contributed to equivalent treatment outcomes in an OA mouse model in terms of histological analysis, radiographic, immunohistological, and molecular analysis results. Furthermore, the amelioration effect of the n‐HA on OA development was partially ascribed to the attenuation of chondrocyte senescence, thereby leading to inhibition of TLR‐2 expression and then blockade of NF‐κB activation. Collectively, the n‐HA may be a promising therapeutic alternative to current commercial HA products for OA treatment.

## 1 INTRODUCTION

Osteoarthritis (OA) is a serious chronic degenerative disease characterized by articular cartilage loss, synovitis, subchondral bone sclerosis, and osteophyte, which is the main cause of joint pain and disability.^1^, ^2^, ^3^, ^4^, ^5^, ^6^ The progression of OA may ultimately lead to keen arthroplasty surgery to restore joint function.^7^, ^8^ Although there are no effective disease‐modifying therapies available to delay or prevent knee replacement, intra‐articular therapies such as corticosteroids (CS), nonsteroidal anti‐inflammatory drugs (NSAIDs), and hyaluronic acid (HA) have been commonly used in clinical trials to mitigate cartilage destruction and knee pain.^9^, ^10^, ^11^ However, the prolonged use of CS may result in chondrocyte apoptosis while the frequent injection of NSAIDs may cause adverse effects on liver and kidney functions.^12^ Compared to the healthy group, the OA patients have less HA with lower quality in their knee joint cavities, thereby leading to higher friction due to the reduced viscoelasticity of synovial fluid.^13^ Therefore, injection of HA viscosupplements is one of the most recommended strategies for pain relief in patients suffering from mild‐to‐moderate OA in clinics.^14^ Because of the existence of hyaluronidase and reactive oxygen species (ROS) in human body, the routine therapeutic mode of current commercial HA products requires four injections monthly for effective relief of knee pain, thus easily causing inconvenience for patients especially in the COVID‐19 pandemic and also pain at the injection site.^15^, ^16^, ^17^ In addition, frequent injection may increase the chance of joint infection.^18^ To improve the enzymatic or ROS resistance of HA and reduce the risk of infection from multiple injections, an attractive strategy is to cross‐link HA through specific chemical reactions to generate HA hydrogel systems.^19^, ^20^, ^21^, ^22^ For example, chemical reagents, such as divinylsulfone (DVS), glycidyl methacylate (GMA), and butanediol diglycidyl ether (BDDE), are commonly adopted to attain chemically crosslinked HA with similar molecular weight to endogenous HA.^23^, ^24^ However, clinical concerns have been raised on the biosafety of these cross‐linking agents. For example, the residual cross‐linking agent in joints may cause severe inflammatory symptoms including joint swelling and redness, local pressure, skin rash, vomiting, and fever.^18^ Moreover, since the HA cross‐linking process may impair the injectability of HA materials, how to balance the injectability and resistance to enzymatic or ROS degradation of HA‐based hydrogels is thus another critical concern.

Granular hydrogels, also known as densely packed or jammed hydrogel microparticles, have recently contributed significantly to advances in material chemistry and tissue engineering because of their unique properties including inherent porosity, shear‐thinning, self‐healing, modular nature, and so on.^1^, ^4^, ^25^ Inspired by this, we reported a novel BDDE‐crosslinked HA granular hydrogel (n‐HA) for the first time as a promising therapeutic alternative to current commercial HA products for OA treatment. First, the biocompatibility and bioefficacy of n‐HA were systematically investigated to test its potential as a viscous supplement for OA patients through a series of in vitro and in vivo experiments. Then, the potential mechanism behind n‐HA attenuating OA development was uncovered. We found that n‐HA can block TLR‐2/NF‐κB signaling pathway by inhibiting cellular senescence, thereby exerting anti‐inflammatory and cartilage protective effects. This work may layout the foundation for fundamental and translational research on potential therapies for OA.

## 2 MATERIALS AND METHODS

### 2.1 Preparation of n‐HA

First, sodium hyaluronate powder (1.2–1.3 × 10^6^ Da from Shandong Zhongshan Biotechnology Co., Ltd., Shandong, China) was dissolved in 1% NaOH by vigorous stirring to prepare 13.3 wt% HA solution. After the addition of 0.5, 0.8, 1, or 1.5 vol% 1,4‐butanediol diglycidyl ether (BDDE: *ρ* = 1.1 g/ml, purity ≥ 95% from Aladdin Co., Ltd., Shanghai, China) into the HA solution, the mixture was then emulsified with the fluorinert™ FC‐40 oil containing 2 wt% Pico‐Surf™ as the oil phase. Afterwards, the mixture was kept at 40°C for 4–6 h to obtain the HA hydrogel microparticles. The hydrogel microparticles were further screened by different mesh screens to collect products with sizes ranging from 100 to 150 μm. Then, the hydrogel microparticles were purified with 2‐(perfluorohexyl)ethyl alcohol and saline. n‐HA was finally obtained at a concentration of 10 mg/ml by centrifugation to remove excess saline and used for the following experiments. The nomenclature of the various n‐HAs discussed in this study was achieved using the volume fraction of BBDE applied in the synthetic steps as the n‐HA prefix. For example, 0.5% n‐HA corresponds to the n‐HA sample composed of hydrogel microparticles synthesized using 0.5 vol% BDDE.

### 2.2 Characterization of n‐HA

#### 2.2.1 Morphology characterization of the granular gel after injection

Morphology characterization of n‐HA after injection was carried out by an inverted microscope (BDS400; Conptec, China) and a confocal microscopy (LSM710; Zeiss, Germany). For inverted microscopy characterization, n‐HA was directly dispersed in saline and observed. Moreover, n‐HA was dispersed in saline containing tetramethylrhodamine isothiocyanate‐dextran (20 kDa, SIGMA) to detect the interstitial space using confocal microscopy.

#### 2.2.2 Nuclear magnetic resonance and Fourier‐transform infrared spectroscopy

^1^H NMR spectra (400 MHz) of HA, 0.5% n‐HA, 0.8% n‐HA, 1% n‐HA, and 1.5% n‐HA in D_2_O were analyzed by a Bruker ACF‐400 spectrometer. Since n‐HA is composed of crosslinked hydrogel microparticles, which are poorly soluble in water, n‐HA was completely digested using hyaluronidase (Aladdin, 300 U/ml). Afterward, the supernatant was collected through centrifuge and then lyophilized and redispersed in D_2_O for ^1^H NMR analysis. Fourier‐transform infrared (FTIR) of HA, 0.5% n‐HA, 0.8% n‐HA, 1% n‐HA, and 1.5% n‐HA were recorded employing a Nicolet 6700 spectrometer (Thermo, USA) from the wave numbers 4000 to 400 cm^−1^.

#### 2.2.3 Injectable capacity and morphology of n‐HA

The n‐HA was aspirated into a syringe with 25 G needle to test their injectable capacity. The cross‐sectional morphology of freeze‐dried n‐HA was examined by scanning electron microscopy (SEM) (Quanta 400F; Philips, Netherlands).

#### 2.2.4 Rheological properties and biodegradability

The rheological properties of n‐HA and the commercial HA product (ARTZ®, Japan, 10 mg/ml) were measured using the Haake Mars 40 Rheometer with parallel plate geometry (8 mm flat plate). All rheological tests were performed at 25°C. Specifically, amplitude sweep tests were carried out with oscillation strain amplitudes of 0.1%–1000% at a constant angular frequency of 1 Hz. The frequency sweep tests were performed in the angular frequency range of 0.1–100 rad/s at a constant strain amplitude of 1%.

Enzymatic degradation experiments of n‐HA and ARTZ® were performed according to previously published protocols.^26^, ^27^ In brief, 2 g of n‐HA or ARTZ® was added to 2 ml solution (30 mM citric acid, 150 mM Na_2_HPO_4_, 150 mM NaCl, pH 6.3) supplemented with 300 U/ml hyaluronidase (HAase) at 37°C. The 100 μl of the supernatant was taken at different time intervals, and the content of uronic acid was determined by the carbazole method to determine the degradation rate of the sample.^26^

ROS‐induced degradation rate of n‐HA was determined using a gravimetric method. Specifically, newly formed 0.5%, 0.8%, and 1.0% n‐HA samples were weighed (M_0_) and immersed in PBS with 0.53 M hydrogen peroxide (H_2_O_2_, Sinopuncture Chemical Reagents Co., Ltd) at a concentration of 33.3 mg/ml. At specific time intervals, after excess solution was removed by centrifugation at 10,000 RPM for 10 min, the remaining n‐HAs were weighed (M_r_). The degradation ratio was expressed as (M_r_/M_0_) × 100%. All experiments were performed in triplicate.

### 2.3 Primary chondrocyte isolation and culture

Primary chondrocytes were isolated from knee articular cartilage of 1‐week‐old C57B/L6 male mice with the permission by the Animal Experimental Ethics Committee of Sun Yat‐sen University. Briefly, the articular cartilage was cut into small pieces (1 × 1 × 1 mm^3^), then washed with PBS (GIBCO, C10010500BT) for three times prior to collection in a centrifuge tube. Afterward, the dissected cartilage pieces were digested in 0.2% collagenase II for 4 h at 37°C. The collected chondrocytes were cultured in DMEM/F12 (Procell, PM150312‐500) medium supplemented with 10% FBS (GIBCO, 10099‐141) and 1% penicillin–streptomycin (GIBCO, 15140122) in an incubator maintained at 5% CO_2_ and 37°C.

### 2.4 Cell cytotoxicity

The primary chondrocytes were seeded in a 96‐well plate at a density of 5 × 10^3^ cells/well and cultured overnight prior to the addition of a series levels (0.2–5 mg/ml) of n‐HA or ARTZ®. After 24 h, cytotoxicity assessment was conducted using the Cell Counting Kit‐8 (CCK‐8, GLPBIO, GK10001, USA) assay.

In addition, the cytocompatibility of n‐HA and ARTZ® was assessed by live and dead staining assay. In a nutshell, the isolated chondrocytes at passage one or two were seeded in a 48‐well plate at a density of 1 × 10^4^ cells/well and cultured overnight prior to the addition of 1 mg/ml of n‐HA or ARTZ®. After 1, 2, and 3 days, the viability of the cells was analyzed using the Live/Dead cell‐mediated cytotoxicity kit (US Everbright Inc.).

### 2.5 Construction of cell inflammation model and cell senescence model

In order to construct a cellular inflammation model, we used TNF‐α (Affinity Biosciences, BF0170‐100) with 20 ng/ml to culture chondrocytes for 24 h (Figure S1). In addition, normal chondrocytes were stimulated with TNF‐α (10 ng/ml) for 48 h to induce chondrocyte senescence, thereby constructing an in vitro cell senescence model.^28^

### 2.6 Phalloidin staining

The chondrocytes were seeded in laser confocal dish at a density of 2 × 10^4^ cells/dish and cultured overnight. The 1 mg/ml of n‐HA or ARTZ® was then added in the presence of TNF‐α with 20 ng/ml. After 24 h, cells were incubated with red phalloidin fluorescent working solution (Abcam, USA) for 60 min according to the method described in the instruction and then incubated with DAPI dye for 5 min. Confocal laser scanning microscopy (CLSM) (FV3000; Olympus) was used to observe cell morphology.

### 2.7 Cell apoptosis

Apoptotic cells were detected by the Annexin V‐FITC/propidium iodide (PI) apoptosis detection kit (KeyGEN BioTECH, China). First, primary chondrocytes were seeded in a six‐well plate at a density of 1 × 10^5^ cells/well and cultured overnight. The 1 mg/ml of n‐HA or ARTZ® was then added in the cell culture medium with or without supplementation of TNF‐α with 20 ng/ml. After 24 h, the suspended chondrocytes after enzymatic digestion were washed twice with PBS and then stained with Annexin V‐FITC for 10 min at room temperature, followed by PI staining for another 5 min. After that, the cells were analyzed by flow cytometry (BD, USA). The number of events recorded in the sample was 10,000 cells.

### 2.8 Senescence‐associated‐β‐galactosidase staining assay

Normal chondrocytes were stimulated with TNF‐α (10 ng/ml) for 48 h to induce chondrocyte senescence, thereby constructing an in vitro cell senescence model. Cellular senescence was then measured using a senescence‐associated‐β‐galactosidase (SA‐β‐Gal) assay kit (Beyotime, China). The senescent cells were seeded in a 96‐well plate at a density of 5 × 10^3^ cells/well and cultured overnight. The 1 mg/ml of n‐HA or ARTZ® was then added into the cell culture medium. After 24 h, the cells were washed twice with PBS after aspiration of the culture medium. Afterward, the cells were fixed by the SA‐β‐Gal staining fixative for 15 min at room temperature. After that, the cells were washed with PBS for three times. Finally, the staining working solution was added for incubation overnight at 37°C in an incubator. The number of SA‐β‐Gal positive cells from three random fields was recorded for calculation of the average counts.

### 2.9 Safranin‐O staining of chondrocytes in vitro

Primary chondrocytes were seeded in a six‐well plate at a density of 5 × 10^4^ cells/well and incubated overnight for adhesion. Afterward, Safranin‐O staining (Roles‐Bio, China) was conducted in TNF‐α with 20 ng/ml induced chondrocytes with or without n‐HA treatment.

### 2.10 Quantitative real‐time polymerase chain reaction analysis

Total RNA was extracted from cultured chondrocytes or articular cartilage using RNA Extraction Kit (Qiagen, Germany). cDNA was transcribed from RNA samples by using reverse transcription reagents (Roche, China) and Real time quantitative polymerase chain reaction assays were carried out to quantify the levels of mRNA expression of these genes. The primers used in this work were designed by Sangon Biotech Co., Ltd. (Shanghai, China), and the sequences of these primers are listed in Table S1.

### 2.11 Western blotting

OA‐related protein expression in chondrocytes was detected by Western blotting. Briefly, protein extracted from chondrocytes were separated by SDS‐PAGE and transferred to PVDF membrane. Membranes were blotted with primary antibodies recognizing TLR‐2 (Abcam, USA), NF‐κB p65 (Bioss, China), NF‐κB phospho‐p65 (Bioss), and GAPDH (Bioss), respectively. All blots were probed with horseradish peroxidase (HRP)‐conjugated secondary antibodies (Zen Bioscience, China) and revealed using the enhanced chemiluminescence (Millipore, USA) detection system.

### 2.12 Animal model

All the operations were performed with the approval by the local Animal Care and Use Committee (SYSU‐IACUC‐2020‐000328). Thirty 8–12 weeks male C57BL/6 mice were purchased and housed in a specific‐pathogen‐free animal facility. Mice were divided into five groups randomly. Destabilization of the medial meniscus (DMM) surgery was conducted in mice to induce OA model. After 2 weeks, according to treatment instruction in clinics, a total volume of 10 μl of saline, n‐HA (10 mg/ml), or ARTZ® (10 mg/ml) was intra‐articularly injected into the mice with a microinjector. The saline and ARTZ® were administrated for 4 consecutive weekly intra‐articular injections while n‐HA was conducted for only one single injection during a course of treatment.

### 2.13 Micro‐CT analysis

The potential changes of subchondral trabecular bone microarchitecture and mineral density in the region of interest (ROI) beneath subchondral cortical bone were measured by micro‐CT. Briefly, the knee joint of the mouse was isolated after euthanasia, and the surrounding soft tissue was removed. After fixation with 4% paraformaldehyde solution for 24 h, the knee joints were scanned using a micro‐CT system (Scano Medical μCT 100, Switzerland) with the scanning parameters set as 55 kV, 200 μA, and 10 μm in spatial resolution.

### 2.14 Histological and immunohistochemical analysis

The tissue sections were cut with a thickness of 4 mm along the coronal plane. Cartilage injury was assessed by Safrani O/Fast Green staining and toluidine blue staining. The OARSI OA histopathology assessment system was chosen to assess the cartilage degeneration severity. The articular cartilage thickness was measured by image J, while the proteoglycan content was scored based on the reported modified Mankin scoring system.^29^

For immunohistochemical staining, the sectioned tissue was treated by high temperature in buffers for antigen retrieval prior to incubation in 3% H_2_O_2_ for 15 min to quench endogenous catalase. The blocked sections with serum were then incubated with primary antibodies recognizing TLR‐2 antibody (Abcam, USA), P16^INK4a^ antibody (Bioss), COL2A1 antibody (Bioss), and MMP13 antibody (Bioss), respectively. After being washed three times with PBS and incubated with goat‐anti‐rabbit HRP‐conjugated secondary antibody for 1 h at 37°C, an immunohistochemical staining signal was developed with DAB substrate system. Image J software (National Institutes of Health, MD, USA) was used to quantify and analyze IHC‐positively stained cells.

### 2.15 Statistical analysis

Data are presented as mean ± standard deviation (mean ± S.D). Differences between two groups were statistically analyzed by unpaired, two‐tailed Student's *t* test while analysis of variance (ANOVA) with Dunnett post hoc test was used for the comparison of data in more than two groups of variables. The level of significance was set at *p < 0.05, **p < 0.01, ***p < 0.001. All statistical analyses were performed with GraphPad Prism software version 8.0 (GraphPad Software, Inc., CA, USA).

## 3 RESULTS

### 3.1 Preparation and characterization of n‐HA

**FIGURE 1:**
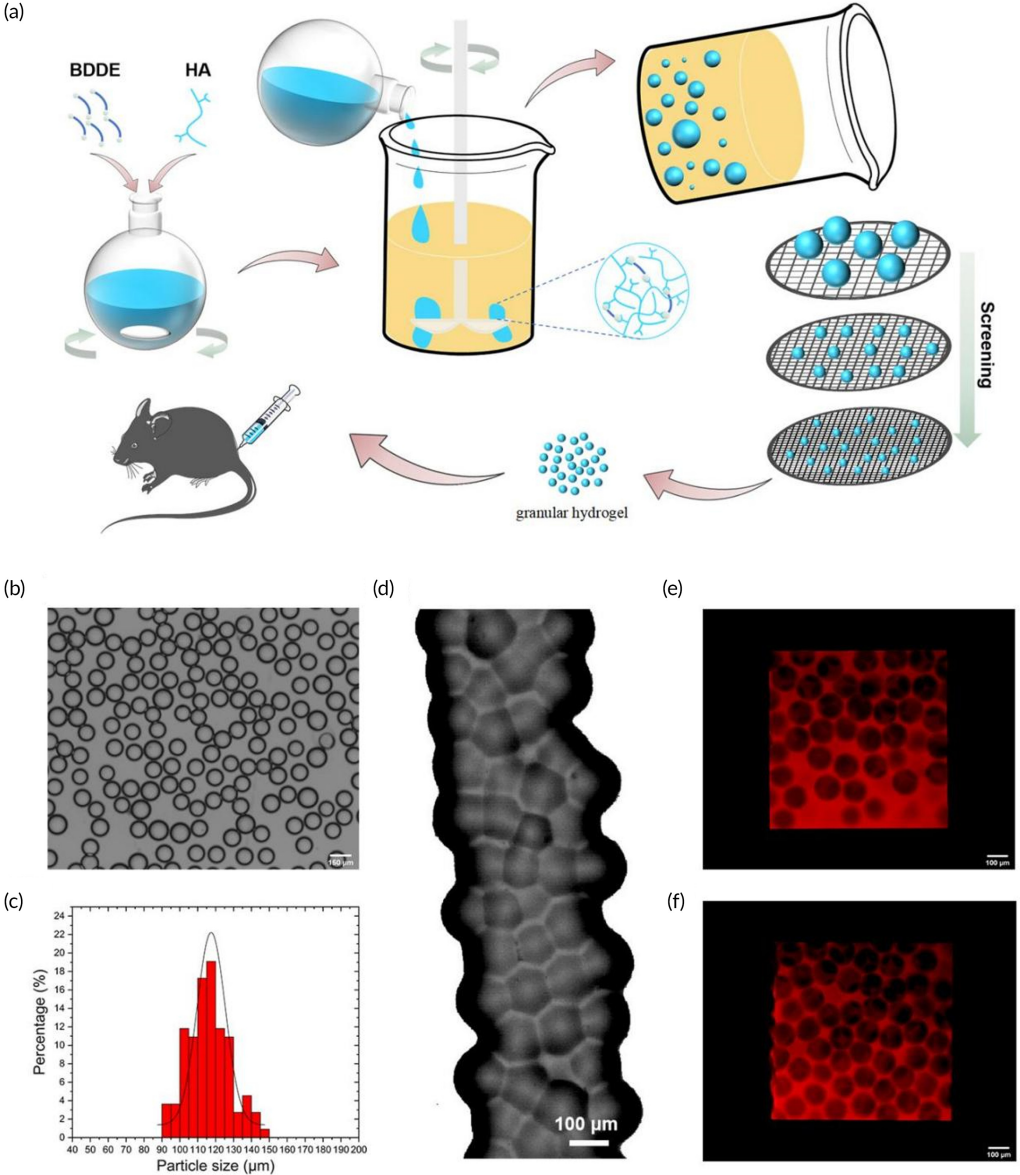
(a) Preparation scheme of granular hydrogel (n‐HA). (b) Microscope images of hydrogel microparticles dispersed in saline. Scale bar = 150 μm. (c) The size distribution of hydrogel microparticles dispersed in saline. (d) Microscopic image of n‐HA. Scale bar = 100 μm. (e, f) Confocal fluorescent microscopic images of n‐HA. Scale bar = 100 μm

**FIGURE 2:**
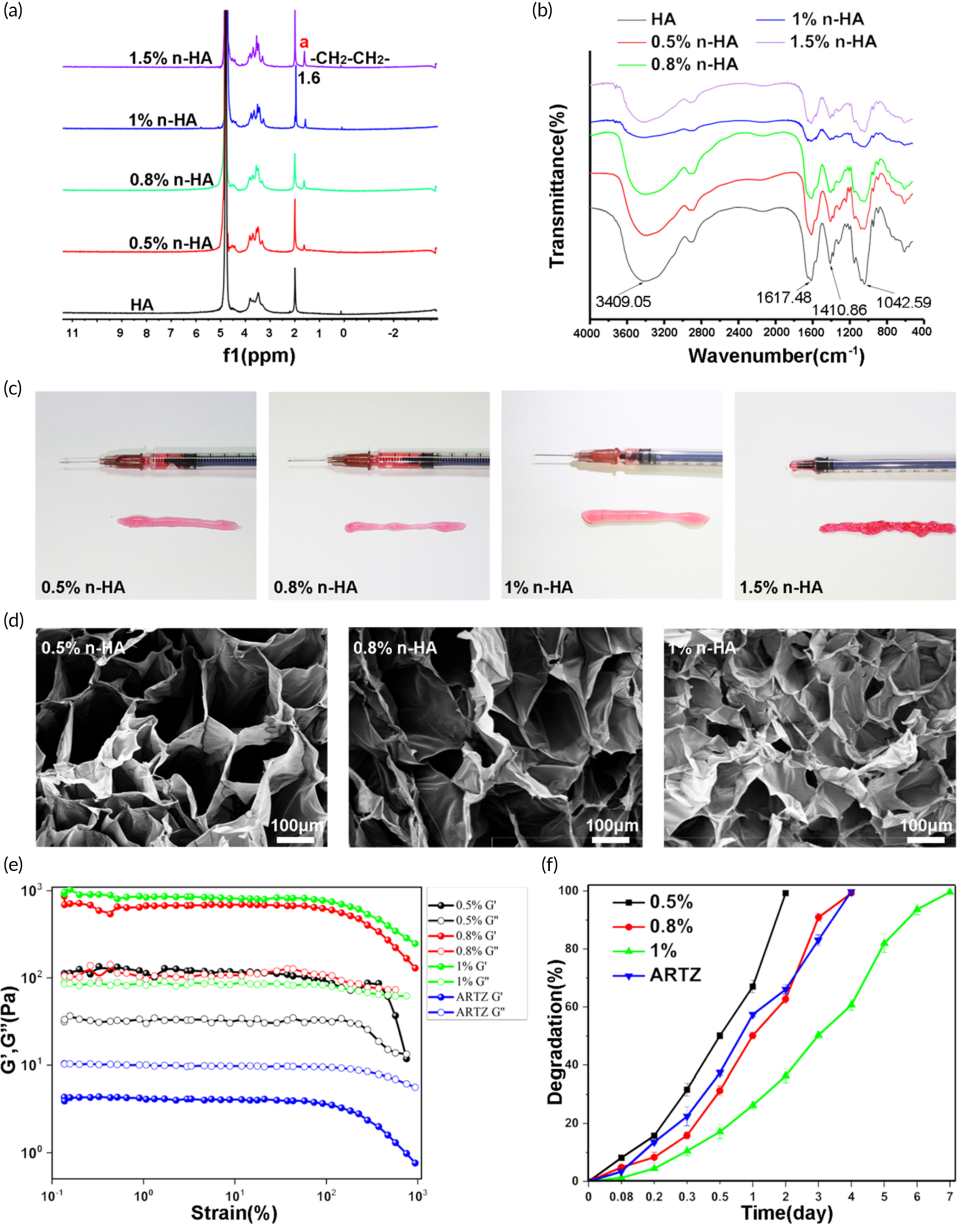
(a) ^1^H‐NMR spectra and (b) Fourier‐transform infrared (FTIR) of hyaluronic acid (HA), 0.5% n‐HA, 0.8% n‐HA, 1% n‐HA, and 1.5% n‐HA (butanediol diglycidyl ether [BDDE] concentrations used to crosslink HA were 0.5, 0.8, 1, and 1.5 vol%, respectively). (c) Injectable capability test of 0.5% n‐HA, 0.8% n‐HA, 1% n‐HA, and 1.5% n‐HA. (d) Scanning electron microscopic images of 0.5% n‐HA, 0.8% n‐HA, and 1% n‐HA. Scale bar = 100 μm. (e) The storage modulus (*G*′) and loss modulus (*G*′′) of 0.5% n‐HA, 0.8% n‐HA, 1% n‐HA, and ARTZ®. (f) The degradation test of 0.5% n‐HA, 0.8% n‐HA, 1% n‐HA, and ARTZ® in the simulated body fluid supplemented with hyaluronidase (*n* = 3)

As shown in Figure 1a, the HA hydrogel microparticles (HMPs) were prepared by cross‐linking HA in water‐in‐oil droplets with BDDE, and the resulting HMPs were screened through sieves of different meshes. The collected HMPs exhibited regular spherical morphology with sizes ranging from 100 to 150 μm (Figure 1b,c). The HMP dispersion was then concentrated by centrifugation and the supernatant was removed to obtain the final granular hydrogel, n‐HA. Moreover, n‐HA can be delivered by injection through a needle without remarkably affecting the morphology and integrity (Figure 1d). 3D confocal fluorescence microscopy revealed micron‐sized interstitial spaces between the granular hydrogels that allowed rapid diffusion of nutrients (Figure 1e,f).

n‐HAs with varying crosslinking degrees were prepared by changing the amount of BDDE. The structures of pristine HA and resulting n‐HAs were verified by the ^1^H NMR spectra (Figure 2a), and the new peak of n‐HAs at δ 1.6 ppm came from —CH_2_—CH_2_— in BDDE, which indicated the successful incorporation of BDDE in n‐HAs. The ratio between the integration area of the *N*‐acetyl signal (CH_3_) from HA at δ 2.0 ppm and that of the BDDE signals around 1.6 ppm gives the degree of modification (MoD) after correction for the number of protons responsible for each signal (Equation 1) (Figure S2). Accordingly, the MoD of 0.5%, 0.8%, 1%, and 1.5% n‐HA samples was calculated to be 11%, 14.75%, 19%, and 25.75%, respectively (Table S2).

(1) $$\mathrm{MoD}\left(\%\right)=\left(I^{\mathrm{\delta H}1.6}/4\right)/\left(I^{\mathrm{\delta H}2.0/3}\right)*100$$

FTIR spectra of lyophilized HA and n‐HA samples were depicted in Figure 2b, which showed similar characteristic peaks for all the samples. Afterward, the manual injectability of n‐HAs was evaluated. As Figure 2c reveals, 0.5%, 0.8%, and 1% n‐HAs could be aspirated and injected very smoothly while 1.5% n‐HA showed much higher injection resistance. Therefore, only 0.5%, 0.8%, and 1% n‐HA were used in subsequent experimental studies. SEM images showed that the n‐HA pore sizes decreased with the increase of BDDE addition during the synthesis of hydrogel microparticles (Figure 2d). Rheological tests indicated that the storage modulus (*G*′) of n‐HA is higher than the loss modulus (*G*′′), indicating the dominance of elastic nature over viscous nature for n‐HA. Different from n‐HA, ARTZ® exhibited predominantly viscous liquid properties (Figure 2e and Figure S3).

Then, the degradation behavior of n‐HA and ARTZ® by peroxidative cleavage or hyaluronidase was evaluated. As shown in Figure 2f, the complete degradation time of ARTZ® was 4 days, while that of 1% n‐HA was 7 days, indicating that BDDE cross‐linking endows n‐HA with good hyaluronidase resistance. Similar results were also noticed for the degradation of n‐HA by peroxidative cleavage (Figure S4). Consequently, compared to ARTZ®, 1% n‐HA showed improved resistance to degradation and was therefore selected in the following in vitro and in vivo tests.

### 3.2 n‐HA inhibits inflammation‐induced chondrocyte apoptosis and necrosis without causing cytotoxicity

**FIGURE 3:**
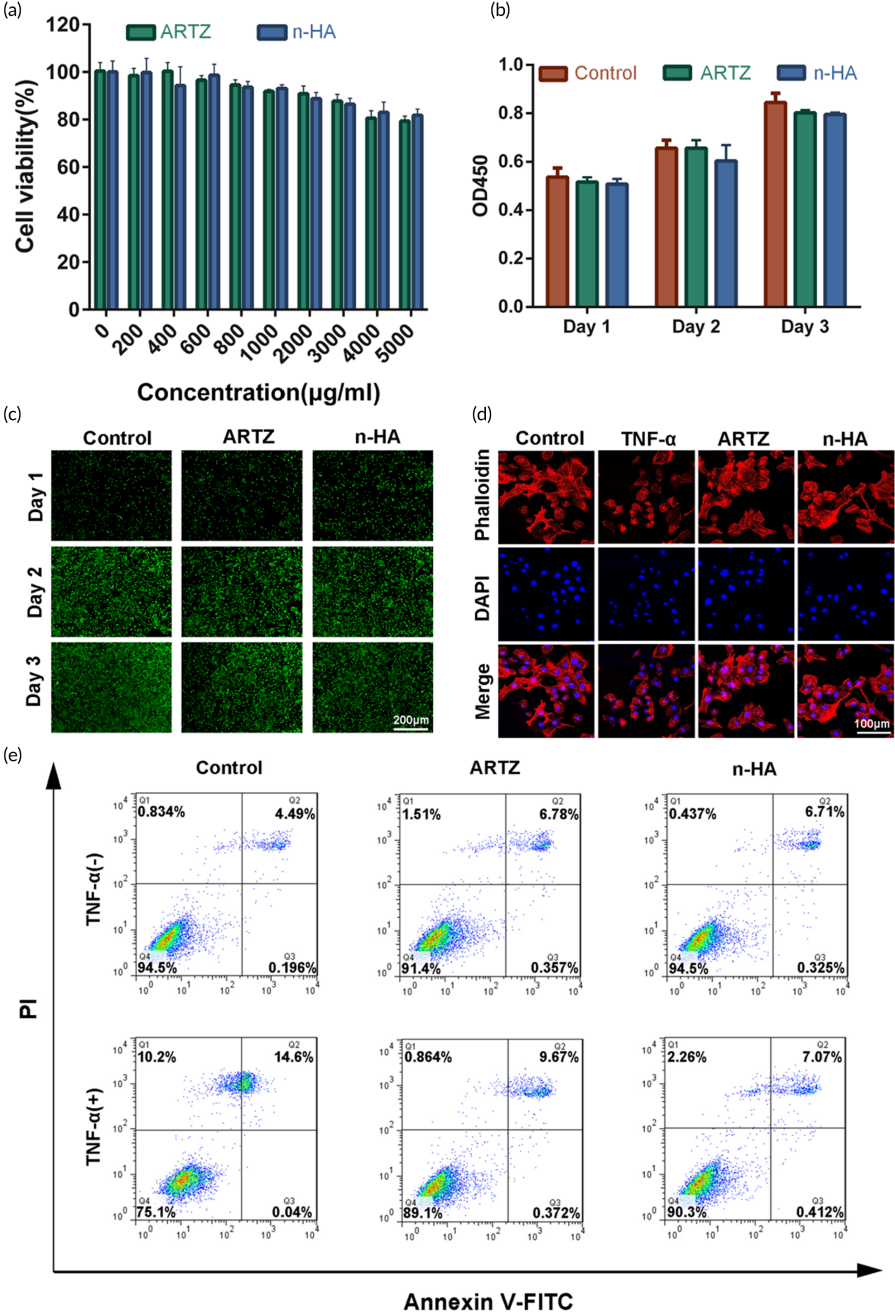
(a) Cell viability of chondrocytes after incubation with different concentrations of ARTZ® and novel HA (n‐HA) using the CCK‐8 assay. (b) Cell proliferation measurement of chondrocytes after incubation with 1 mg/ml ARTZ® and n‐HA for 1, 2, and 3 days using CCK‐8 assay. (c) Live/Dead staining of chondrocytes after incubation with 1 mg/ml ARTZ® and n‐HA for 1, 2, and 3 days, the live cells were stained green, whereas the dead cells were stained red. Scale bar = 200 μm. (d) Representative confocal images of phalloidin stained chondrocytes with or without TNF‐α, ARTZ®, and n‐HA treatment. Scale bar = 100 μm. (e) The quantification of apoptotic rates of chondrocytes with various treatment by flow cytometry. (*n* = 3, *p < 0.05, **p < 0.01, and ***p < 0.001, NS: no significance.)

The cytocompatibility of n‐HA was evaluated by using primary chondrocytes via cell viability and live/dead staining assay. First, we prepared a standard medium containing hydrogel (5 mg/ml) by adding the stock solution (10 mg/ml hydrogel) into 2× concentrated cell culture medium with the same volume. The standard medium can be further diluted with the cell culture medium. Afterward, FBS and penicillin–streptomycin were supplemented into the medium for cell culture prior to the following compatibility testing. After 24 h culture in the presence of n‐HA or ARTZ® with varying concentrations, the cell survival rate was still above 80% for both n‐HA and ARTZ® groups even with 5 mg/ml (Figure 3a), indicating their satisfactory cytocompatibility. Then, the observation time points were set as 1, 2, and 3 days for assessment of cell proliferation in both n‐HA and ARTZ® groups with the fixed concentration at 1 mg/ml (Figure 3b). Encouragingly, the live/dead staining assay also revealed that the addition of n‐HA did not cause extra increase in the number of dead cells (Figure 3c).

As inflammatory responses provoked chondrocyte apoptosis, leading to cartilage destruction in OA patients, TNF‐α treatment in chondrocytes was performed to mimic in vivo microenvironment. As shown in Figure 3d, chondrocyte cell morphology remarkably changed and shrank into a round shape from a polygonal shape with a decreased size. Interestingly, the addition of ARTZ® or n‐HA reversed the morphological perturbations and effectively restored the distribution of clear clusters at the cell boundary in chondrocytes. In addition, the flow cytometry analysis was conducted to evaluate the potential effects of ARTZ® or n‐HA on prevention of chondrocyte apoptosis in the presence of TNF‐α. As shown in Figure 3e, the addition of n‐HA or ARTZ® into the completed cell culture medium did not remarkably cause extra cell apoptosis and necrosis in the absence of TNF‐α. The total necrosis and apoptosis rates of the primary chondrocytes were 5.5%, 8.6%, and 7.5% for the control group, the n‐HA group, and the ARTZ® group, respectively. The treatment of TNF‐α dramatically increased the rate of apoptosis and necrosis up to 24.9%, while the addition of n‐HA or ARTZ® effectively reduced the inflammation induced cell apoptosis and necrosis in chondrocytes. As depicted in Figure 3e, the apoptosis and necrosis rate of chondrocytes treated by TNF‐α was only 9.7% in n‐HA and 11.9% in ARTZ®, respectively, indicating that n‐HA may be a superior viscosupplement to ARTZ® in inhibition of chondrocyte apoptosis and necrosis.

### 3.3 n‐HA exerts anti‐inflammatory ability through attenuating cellular senescence

**FIGURE 4:**
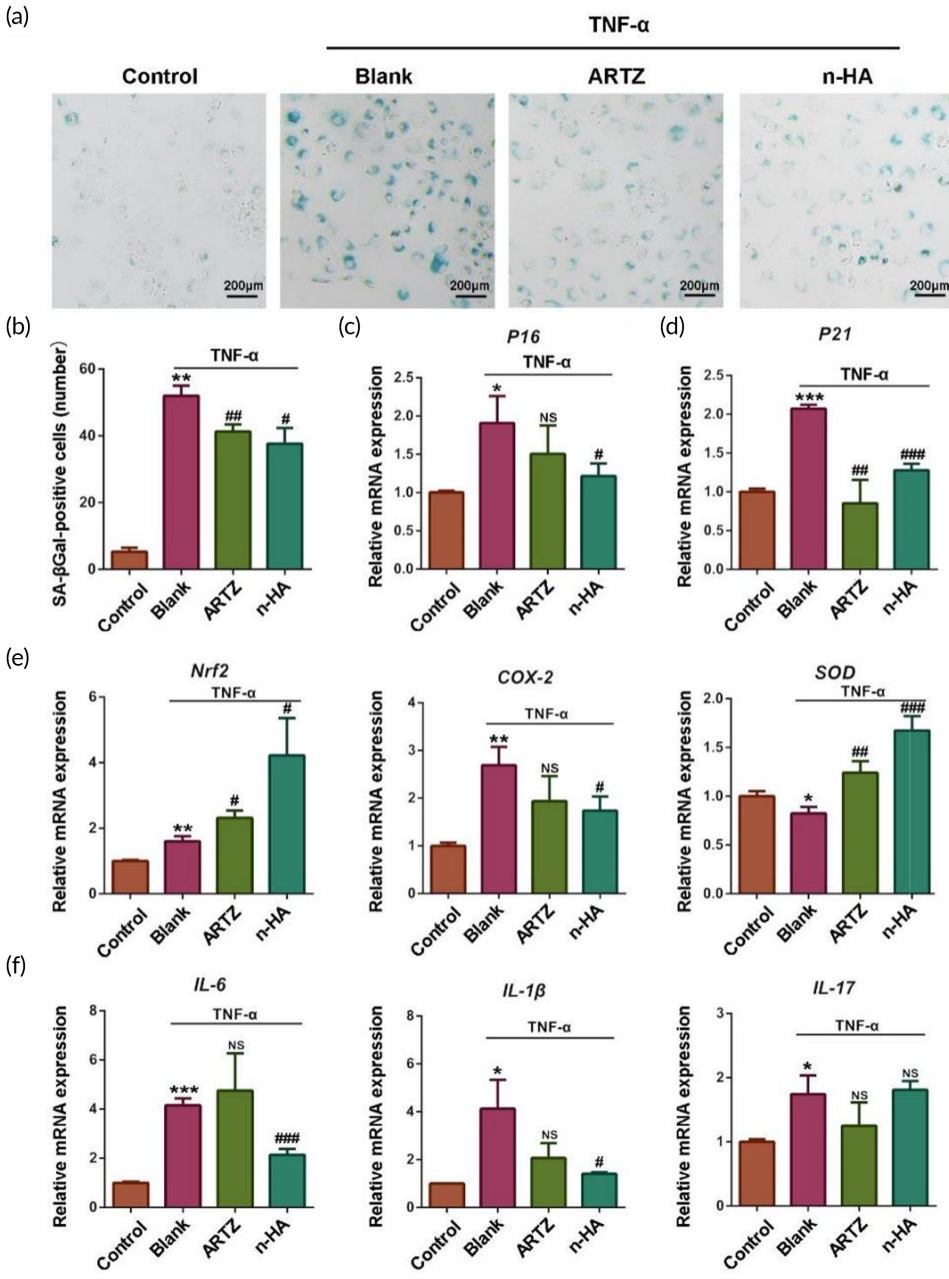
(a) Representative images showing chondrocytes in different groups after SA‐β‐Gal staining. Scale bar = 200 μm. (b) Quantitative analysis of SA‐β‐Gal positive chondrocytes in different groups. Quantitative analysis of mRNA expression levels of p16^INK4a^ (c) and p21 (d) of chondrocytes with or without TNF‐α treatment through RT‐qPCR assay. RT‐qPCR analysis showing the mRNA expression levels of oxidation related genes including Nrf2, COX‐2, and SOD (e), as well as inflammation‐related genes including IL‐6, IL‐1β, and IL‐17 in TNF‐α‐treated chondrocytes (f). (*n* = 3, *p < 0.05, **p < 0.01, and ***p < 0.001, compared with the control groups; #*p* < 0.05, ##*p* < 0.01, and ###*p* < 0.001, compared with the blank groups; NS, no significance)

Most recently, prevention of chondrocyte senescence or local clearance of senescent chondrocytes in articular cartilage has been considered as a promising therapeutic strategy against OA.^30^ The secreted senescence‐associated secretory phenotypes (SASP) by chondrocytes seriously impairs cartilage homeostasis, so the senescence markers, such as SA‐β‐Gal, p16^INK4a^, and p21, were measured and compared in TNF‐α induced chondrocytes with various treatment to test whether n‐HA ameliorated cartilage destruction via modulation of cellular senescence. As shown in Figure 4a, SA‐β‐Gal positive chondrocytes were significantly increased in the blank group, while the addition of n‐HA or ARTZ significantly decreased the number of SA‐β‐Gal positive cells (Figure 4b). Similarly, the presence of n‐HA or ARTZ® significantly reversed the increased expression levels of p21 in chondrocytes. Importantly, compared to ARTZ®, n‐HA showed promoted role in inhibition of p16INK4a expression levels (Figure 4c,d).

Another characteristic of cellular senescence is evidenced by increased oxidative stress. Nrf2, a key transcription factor that regulates redox reactions and signal transduction, closely mediates expression of oxidative stress markers including COX‐2 and SOD, thereby contributing to modulation of the progression of inflammatory disorders.^31^ As shown in Figure 4e, the gene expression level of Nrf2 was significantly increased in senescent cells. It is of note that the addition of n‐HA or ARTZ® significantly promoted Nrf2 gene expression level in chondrocytes. In addition, when compared to ARTZ®, n‐HA showed significantly improved ability in inhibition of COX‐2 while promotion of SOD expression levels, respectively. Thus, no matter ARTZ® or n‐HA significantly inhibited oxidative stress in the TNF‐α induced chondrocytes relative to the blank medium. Exposure to exogenous TNF‐α directly provoked SASP generation as well as oxidative stress production in chondrocytes, thereby leading to initiation of inflammatory responses. Then, the gene expression levels of pro‐inflammatory cytokines including IL‐6, IL‐1β, and IL‐17 were measured in chondrocytes with various treatment. As shown in Figure 4f, n‐HA significantly reduced the expression levels of IL‐6 and IL‐1β in TNF‐α treated chondrocytes relative to ARTZ®. Interestingly, IL‐17 expression levels of TNF‐α‐treated chondrocytes did not show remarkable changes with the addition of ARTZ® or n‐HA. In conclusion, n‐HA can significantly reduce chondrocyte oxidative stress and inhibit inflammatory response by preventing cellular senescence.

**FIGURE 5:**
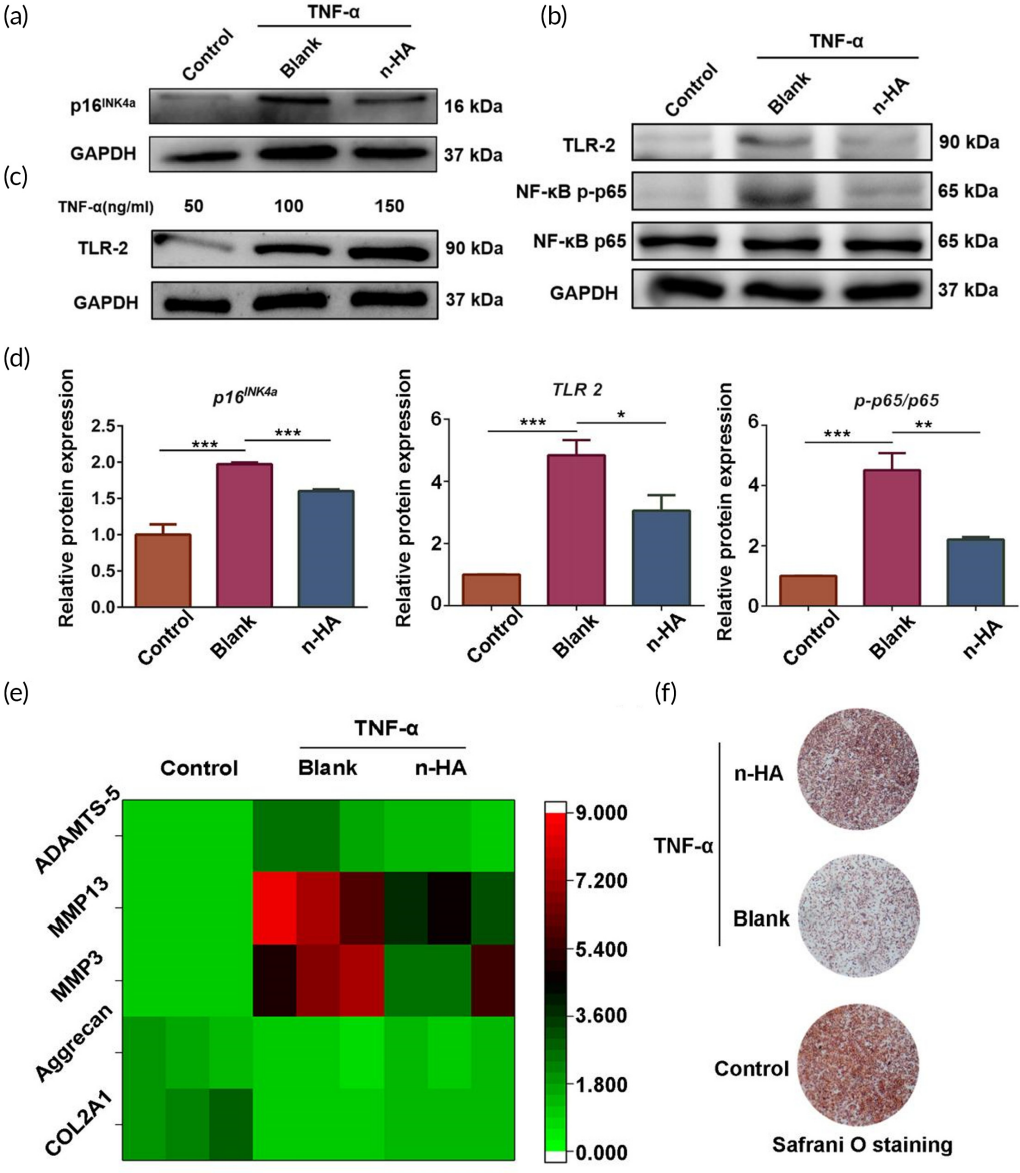
Representative Western blot images (a, b) and summary data (d) showing p16^INK4a^, TLR‐2, NF‐κB p65, and NF‐κB p‐p65 levels in chondrocytes with or without TNF‐α stimulation. (c) Representative images of Western blot for TLR‐2 in chondrocytes induced by TNF‐α with varying concentrations. (e) Heatmap displaying the gene expression levels of COL2A1, Aggrecan, MMP3, MMP13, and ADAMTS‐5 in chondrocytes with or without TNF‐α stimulation. (f) Representative Safrani‐O staining images showing chondrocytes with or without TNF‐α stimulation. (*n* = 3, *p < 0.05, **p < 0.01, and ***p < 0.001; NS, no significance)

The pro‐inflammatory cytokines, secreted via autocrine or paracrine manner in the knee join microenvironment from chondrocytes and synovial macrophages, respectively, trigger chondrocyte apoptosis through activating inflammation involved signaling pathways.^32^ Recently, numerous studies have revealed that toll‐like receptors (TLRs), specifically TLR‐2 and TLR‐4, play a critical role in the pathogenesis of OA through binding to pro‐inflammatory cytokines in chondrocytes and synovial tissue.^33^, ^34^, ^35^ Thus, the inhibition of pro‐inflammatory cytokines and prevention of TLRs dependent signaling pathway might be one of the promising therapeutic directions in OA treatment. Although enormous investigation reported that HA suppressed massive inflammatory mediators through blocking TLRs,^36^ the mechanism underlying the modulation pathway is still uncovered. As shown in Figure 5a we found that n‐HA significantly reduced the protein levels of p16INK4a and TLR‐2 as well as inhibited NF‐κB activation in senescent cells (Figure 5b,d). Notably, TLR‐2 expression level in chondrocytes increased with the dose of pro‐inflammatory cytokines (Figure 5c), implying that SASP secreted by senescent chondrocytes is a potential modulator affecting TLR‐2 level. Together, n‐HA may exert the anti‐inflammation role through suppressing TLR‐2 expression level, which was ascribed to the inhibition of cellular senescence.

As the activation of NF‐κB signaling pathway contributes to upregulation of pro‐inflammatory cytokines, n‐HA treatment may suppress the inflammatory responses by modulating the inflammatory microenvironment. As shown in the heat map (Figure 5e), the gene levels of MMP13, ADAMTS‐5 and COX‐2 dramatically increased in chondrocytes after TNF‐α treatment, while the levels of COL2A1 and Aggrecan remarkably decreased. Of note, the supplementation of n‐HA in the TNF‐α induced chondrocytes effectively reversed inflammation, contributing to reduction of gene expression levels of MMP3, MMP13 and ADAMTS‐5 and increase of COL2A1 and Aggrecan expression levels. Consistently, Safranin‐O staining results exhibited TNF‐α treatment caused much loss of the extracellular matrix (ECM) in chondrocytes while the addition of n‐HA reduced ECM destruction (Figure 5f).

**FIGURE 6:**
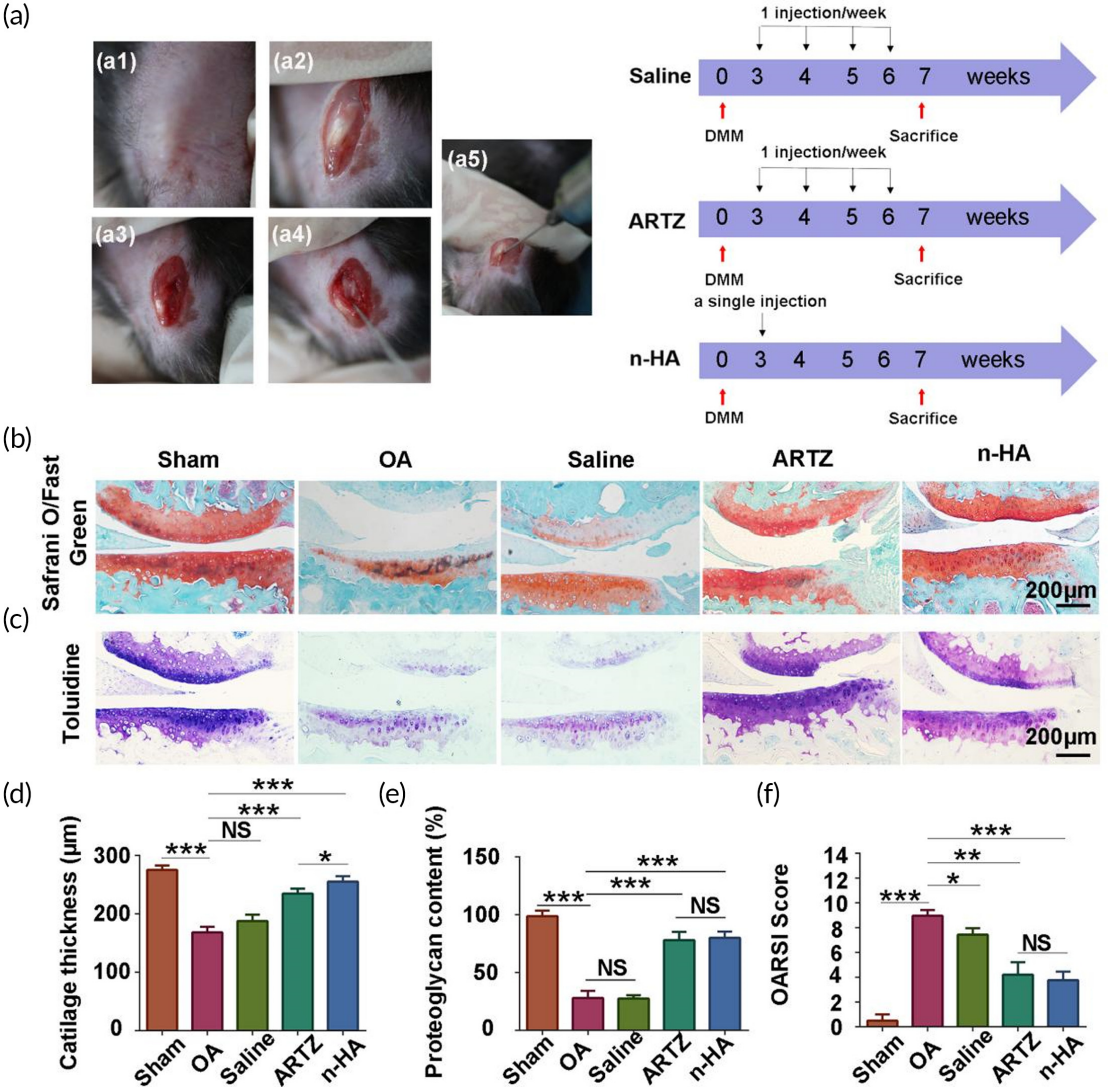
(a) Experimental procedure showing destabilization of the medial meniscus (DMM) surgery performed in mice and intervention protocol using saline, ARTZ®, and novel HA (n‐HA). (a1–a4) DMM surgery model process; (a5) saline, ARTZ® or n‐HA were intra‐articularly administered into animals. (b) Representative images of Safrani‐O/fast green staining of knee joint sections of osteoarthritis (OA) model mice with various intervention. Scale bar = 200 μm. (c) Representative images of toluidine blue staining of knee joint sections of OA model mice with various intervention. Scale bar = 200 μm. Summary results of cartilage thickness (d) and (e) proteoglycan content of knee joint sections of OA model mice with various intervention. (f) Semi‐quantitative analysis employing OARSI scores for histopathology assessment in mice. (*n* = 5, *p < 0.05, **p < 0.01, and ***p < 0.001; NS: no significance)

In order to further explore the therapeutic effects and simultaneously verify the repair mechanism of n‐HA in OA, the OA mouse model was applied via DMM surgery. At 2 weeks post‐surgery, n‐HA, ARTZ® or saline was intra‐articularly injected into knee joint cavity of mice. During the course of treatment, only one single injection for n‐HA while four consecutive injections for ARTZ® or saline was performed in the OA mouse model (Figure 6a). Compared with the sham group, the mice developed cartilage destruction in their knee joint as expected after DMM surgery, evidenced by observation of loss of proteoglycan and cartilage thickness (Figure 6b,c). No matter n‐HA or ARTZ® injection significantly prevented cartilage loss and degeneration (Figure 6d,e). OARSI cartilage scoring evaluation has been widely used for quantitative assessment of OA severity. As shown in Figure 6f, compared with the OA group without treatment, n‐HA or ARTZ® intervention significantly reduced OARSI scores. Among all the treatment groups, n‐HA exhibited best performance in reduction of OARSI scores although there was no significant difference in OARSI scores between n‐HA and ARTZ® groups.

**FIGURE 7:**
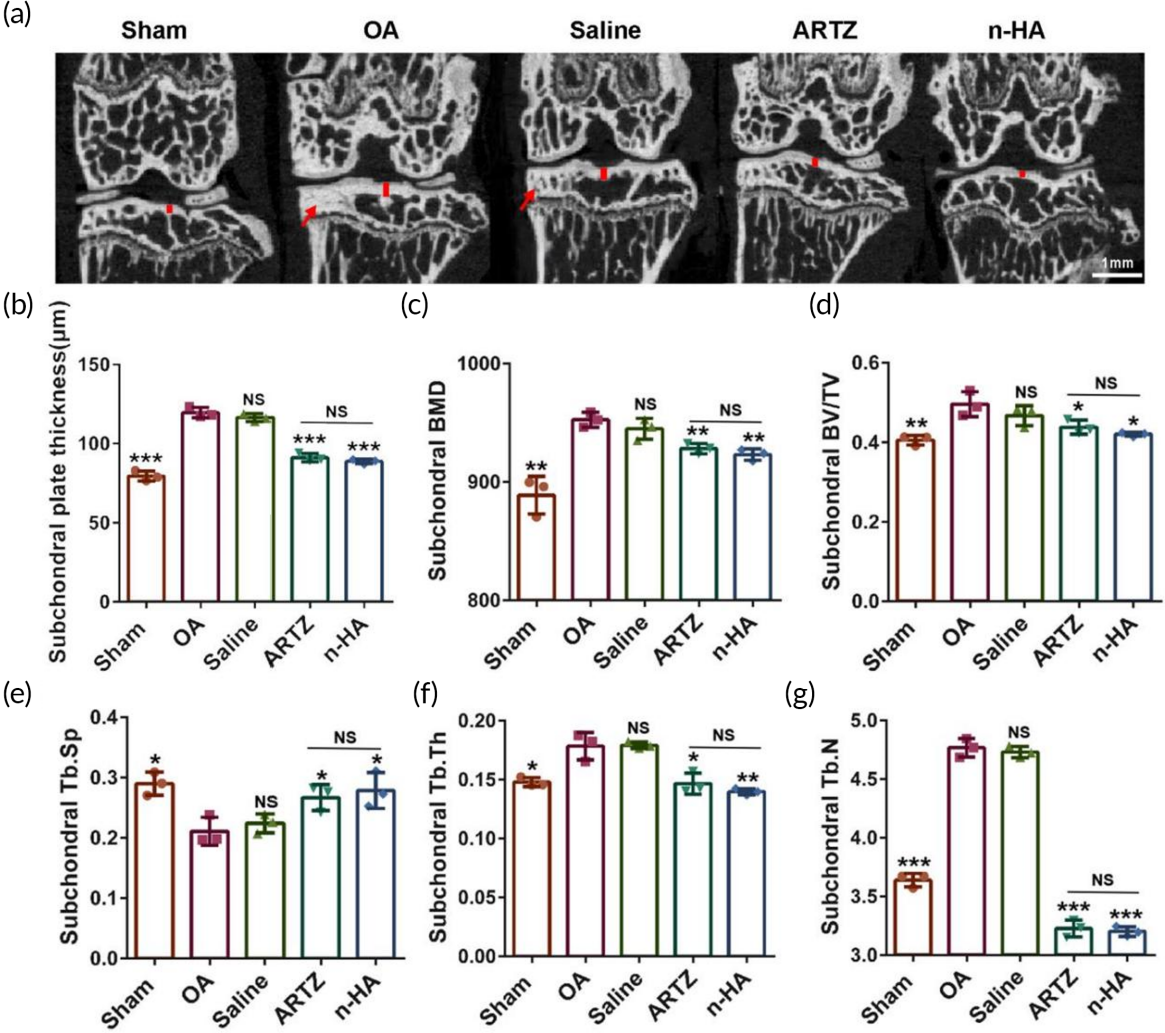
(a) Representative micro‐CT images showing knee joint for subchondral bone sclerosis evaluation. Red arrow indicates subchondral bone sclerosis around the medial tibial plateau; a red line marks subchondral plate thickness. Quantified changes in (b) subchondral bone plate thickness, (c) subchondral bone mineral density (BMD), (d) subchondral bone volume‐to‐total volume ratio (BV/TV), (e) trabecular separation (Trab.Sp), (f) subchondral trabecular thickness (Tb.Th), and (g) trabecular number (Trab.N). (*n* = 3, *p < 0.05, **p < 0.01, and ***p < 0.001, compared with the OA groups; NS: no significance)

Afterward, the quantitative analysis of subchondral bone sclerosis of tibiae in the OA model mice with various interventions was performed by micro‐CT technique. As compared to the sham group, both of the OA group and the saline group showed significantly higher plate thickness (Figure 7a,b), bone mineral density (BMD) (Figure 7c), bone volume density (BV/TV) (Figure 7d), trabecular thickness (Tb.Th) (Figure 7f), and trabecular number (Trab.N) (Figure 7g) in subchondral bone. In addition, both of the OA group and the saline group showed significantly lower trabecular separation (Trab.Sp) (Figure 7e) relative to the sham group in subchondral bone. Encouragingly, these OA‐associated subchondral abnormalities were alleviated by n‐HA or ARTZ® treatment. However, there were no significant differences in these histomorphometric parameters between n‐HA and ARTZ® treated groups (Figure 7a–g).

**FIGURE 8:**
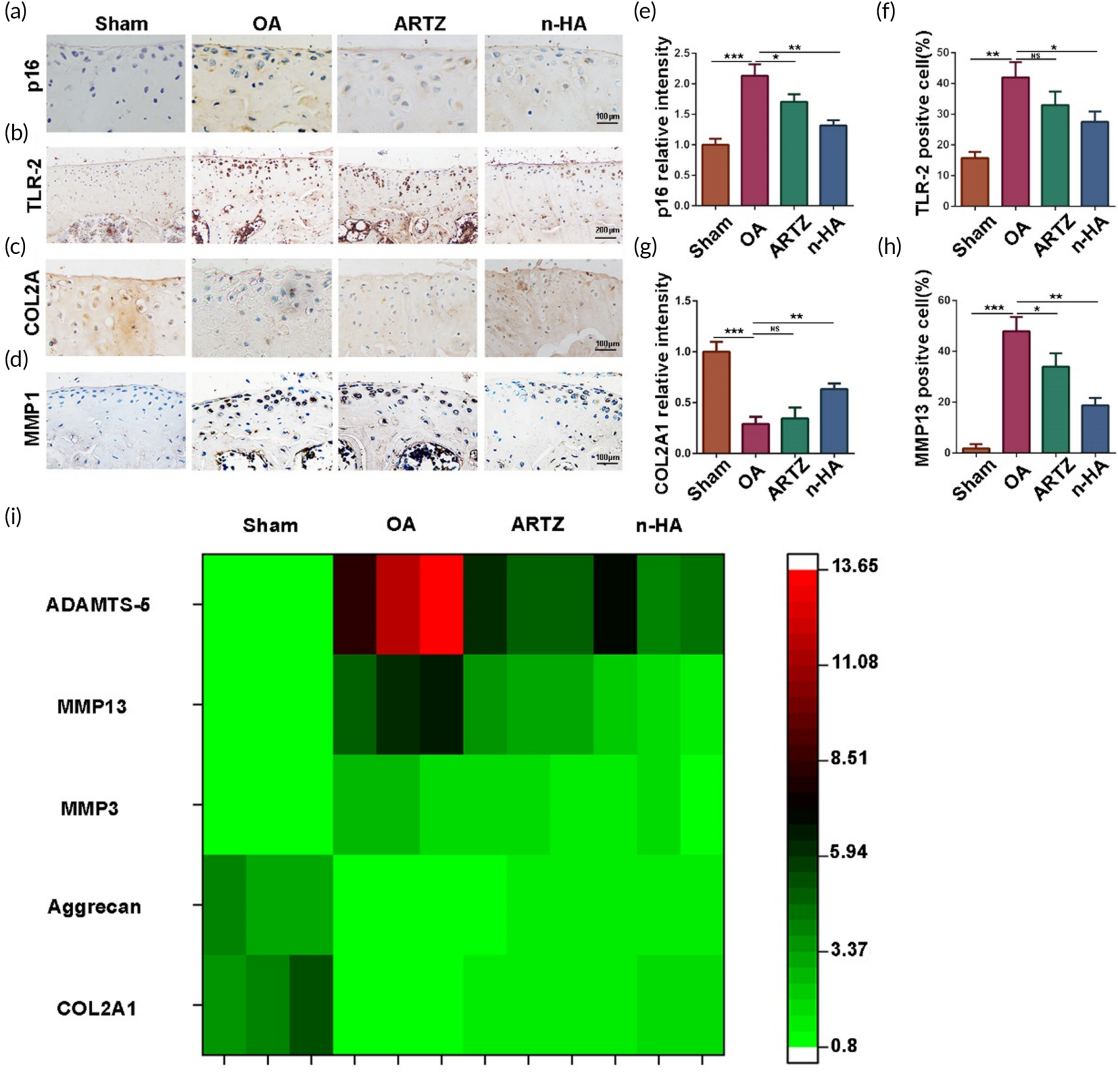
(a–h) Representative immunohistochemistry images and summary data showing P16^INK4a^, TLR‐2, COL2A1, and MMP13 expression in the articular cartilage. (b: scale bar = 200 μm; a, c, d: scale bar = 100 μm). (i) Heat‐map displaying COL2A1, Aggrecan, MMP13, MMP3, and ADAMTS‐5 levels of the articular cartilage harvested from the osteoarthritis (OA) model mice with or without intervention. (*n* = 3, *p < 0.05, **p < 0.01, and ***p < 0.001; NS, no significance)

Afterward, specific markers in the articular cartilage of the OA model mice were identified and quantitatively measured for more solid evidence, which may favor to shed new light on the repair mechanism of n‐HA in prevention of OA progression. First, the p16INK4a senescence marker, serving as a commonly used biomarker of aging, was stained in the articular cartilage of all the mice models. The p16‐expressing chondrocytes in the tibial plateau significantly increased in the OA model mice without intervention when compared to n‐HA or ARTZ® group (Figure 8a,e). Importantly, n‐HA exerted the best performance to inhibit p16INK4a, contributing to high efficiency in clearance of cellular senescence. This finding was in line with our observed results in the TNF‐α induced chondrocytes treated with n‐HA. In addition, n‐HA treatment significantly inhibited TLR‐2 expression levels in the articular cartilage of the OA model mice relative to other intervention methods. Notably, although ARTZ® showed capability in prevention of TLR‐2 expression, there was no significant difference in TLR‐2 expression level of the articular cartilage between ARTZ® and untreated OA groups (Figure 8b,f). Similarly, when compared with the OA mouse without intervention, n‐HA also significantly reduced MMP13 (Figure 8d,h) expression levels in the articular cartilage, ultimately contributing to significant increase of COL2A1 (Figure 8c,g). In addition, RT‐qPCR analysis of the isolated articular cartilage from the OA model mice further convinced that n‐HA remarkably downregulated gene expression levels of MMP3, MMP13 and ADAMTS‐5 while dramatically upregulated COL2A1 and aggrecan levels (Figure 8i). Therefore, both in vitro and in vivo studies revealed that n‐HA and commercial HA product ARTZ® showed equivalent treatment outcomes in OA therapy, which may be ascribed to their unique role in suppression of cellular senescence and then contribute to the blockage of TRL‐2/NF‐κB signaling pathway initiated and modulated by SASP.

## 4 DISCUSSION

Intra‐articular injection of HA viscosupplements is one of the most commonly recommended therapeutic strategies for pain relief and joint lubrication enhancement in mild‐to‐moderate OA patients without clinical safety concerns. However, the exogenous HA supplements rapidly lose their elastoviscosity and anti‐inflammation function in OA patients owing to enzymatic hydrolysis caused by hyaluronidase in human body, leading to frequent injections of HA in one treatment course. Here, a novel HA granular hydrogel with optimized mechanical properties behaved in an elastic‐like manner, contributing to promoted resistance to degradation. Importantly, compared to the ARTZ® commercial HA product with four consecutive injections in one treatment course, n‐HA with one single injection showed equivalent treatment outcome in the OA model mice. Notably, in addition to biosafety and bio‐efficacy assessment, we found n‐HA mitigated OA through attenuation of cellular senescence and subsequent inhibition of TLR‐2/NF‐κB signal pathway.

HA has been investigated extensively in both preclinical and clinical studies and considered as the effective anti‐inflammatory agent to attenuate articular cartilage destruction. However, multiple injections in one treatment course may cause extra economic or mental burden for OA patients especially during outbreak of pandemic, such as current COVID‐19. Therefore, the development of HA with the prolonged retention time in the joint cavity may favor burden relief for both patients and physicians. In addition to our developed crosslinked HA as “one‐shot” injection for pain relief in OA patients, nonchemical modified sodium hyaluronate and hylan G‐F 20 have been reported to show satisfactory clinical outcomes for treatment of OA patients with one single injection.^37^ Most recently, a n‐HA product (Durolane) with very high molecular weight over 100,000 kDa has been tried to treat mild to moderate knee and hip OA patients, revealing beneficial while not significant improved effects.^38^ The controversial clinical outcomes may be caused by injection dose, HA crosslinking degree, OA stages, and criteria. Although HA has been widely used in clinics, the repair mechanism was still lacking. Recent work has found that HA can inhibit synovial inflammation through modulation of GRP78/NF‐κB signaling pathway, contributing to significant reduction of IL‐6 and PGE2 levels.^30^ In addition, HA is negatively correlated with the production of IL‐1β‐stimulated MMPs and free radicals,^39^ thereby mitigating chondrocyte apoptosis and articular cartilage degeneration. Interestingly, HA even showed protective role in tissue‐engineered cartilage in the presence TNF‐α via upregulation of VEGFA and ANKRD37 genes.^40^ Recently, cellular senescence have been viewed as a critical factor influencing cartilage loss and joint inflammation.^30^ As the production of pro‐inflammatory molecules and degenerative enzymes was closely associated with SASP caused by cellular senescence, so local clearance of senescent cells was a promising therapeutic target for attenuation of OA development.^41^ Encouragingly, our developed n‐HA granular hydrogel exhibited the potential in suppression of cellular senescence, contributing to reduced SASP production and thereby inhibiting TLR‐2 levels via a pro‐inflammatory cytokine dose‐dependent manner. Although there have been studies reporting the role of HA in prevention of TLRs for OA treatment, the mechanism underlying the modulation pathway was still not unveiled. Here, we for the first time reported the critical contribution of HA, as the promising anti‐senescence supplement, to prevention of TLRs levels in chondrocytes, ultimately blocking TLR‐2/NF‐κB signaling pathway. In addition, as potential viscosupplements for enhancement of lubrication in OA knee joints, the biocompatibility of n‐HA was systematically validated through a series of in vitro and in vivo experiments. Notably, no extra cytotoxicity or organ toxicity was caused after the use of n‐HA granular hydrogel with the equivalent dose to the commercial HA product (Figure S1).

However, there were some limitations to this study. First, various mild to moderate OA models mimicking different clinical phenotypes in OA patients should be considered to validate the bio‐efficacy of our developed n‐HA granular hydrogel. Then, a longer treatment course may be required for verification of superiority, equivalence or non‐inferiority of n‐HA relative to its commercial counterpart in OA models prior to the potential clinical trials. Although our proof study revealed the advantage of n‐HA with improved resistance to enzymatic hydrolysis, there are still some concerns about the potential phagocytosis of such granular microgels by macrophages. Therefore, it is important to track the fate of n‐HA granular microgels in vivo through signal labeled technology.

## 5 CONCLUSION

We synthesized a n‐HA granular hydrogel for the intervention treatment of OA by adjusting the ratio of BDDE. Compared to the commercial HA product, n‐HA has improved rheological properties and enhanced resistance to enzymatic hydrolysis. In addition, n‐HA with one single injection showed equivalent treatment outcome to the commercial HA product with four consecutive injections in attenuation of OA development. Importantly, n‐HA could alleviate chondrocyte senescence, thereby blocking pro‐inflammatory cytokine dose‐dependent TLR‐2/NF‐κB signaling pathway and ultimately favoring cartilage homeostasis for OA therapy. Taken together, n‐HA, as a biocompatible viscosupplement without inducing cytotoxicity and pathological changes in major organs, may serve as a competitive alternative to current commercial HA counterparts in the treatment of OA patients.

## AUTHOR CONTRIBUTIONS

**Chen Zhang:** Data curation (lead); formal analysis (lead); writing – original draft (equal). **Zhengxiang Cheng:** Data curation (lead); formal analysis (equal); methodology (equal); project administration (equal). **Yuanyuan Zhou:** Data curation (equal); formal analysis (lead). **Ziyi Yu:** Methodology (equal); supervision (equal). **Hongyu Mai:** Methodology (equal); software (equal). **Changhao Xu:** Validation (equal); visualization (equal). **Jing Zhang:** Supervision (lead). **Jiali Wang:** Conceptualization (lead); funding acquisition (lead); supervision (lead); writing – original draft (lead); writing – review and editing (lead).

## CONFLICT OF INTEREST

The authors declare that they have no known competing financial interests or personal relationships that could have appeared to influence the work reported in this paper.

### PEER REVIEW

The peer review history for this article is available at https://publons.com/publon/10.1002/btm2.10475.

## Supporting information


**Table S1:** The specific primers used for different genes
**Table S2:** NMR data
**Figure S1:** Establishment of TNF‐α‐induced inflammatory model. Cell viability of the chondrocytes following incubation with different concentrations of TNF‐α (10, 20, and 30 ng/ml), measured by the CCK‐8 assay. (*p < 0.05, **p < 0.01, and ***p < 0.001).
**Figure S2:** The ^1^H NMR analysis of 1.5% n‐HA.
**Figure S3:** (A) Frequency sweeps of 0.5% n‐HA, 0.8% n‐HA, and 1% n‐HA. The storage (*G*′) and loss (*G*′′) modulus were measured under a constant strain of 1% and angular frequency ranging from 0.1 to 100 rad/s at 25°C. (B) Viscosity of 0.5%, 0.8%, 1% n‐HA and ARTZ as a function of shear rate. (C) The difference in the yield stress of 0.5%, 0.8%, 1% n‐HA and ARTZ. (D) The variation of tan δ with frequency for 0.5%, 0.8%, 1% n‐HA and ARTZ.
**Figure S4:** The degradation test of 0.5% n‐HA, 0.8% n‐HA, and 1% n‐HA under peroxidation (*n* = 3).

## Data Availability

The data that support the findings of this study are available on request from the corresponding author upon reasonable request.
