# Supplementary material for: The novel hyaluronic acid granular hydrogel attenuates osteoarthritis progression by inhibiting the TLR‐2/NF‐κB signaling pathway through suppressing cellular senescence
Source: Bioeng Transl Med. 2022 Dec 23;8(3):e10475. doi: 10.1002/btm2.10475 (PMC10189429; doi:10.1002/btm2.10475)
Supplement: Supplementary file 1 — Table S1: The specific primers used for different genes Table S2: NMR data Figure S1: Establishment of TNF‐α‐induced inflammatory model. Cell viability of the chondrocytes following incubation with different concentrations of TNF‐α (10, 20, and 30 ng/ml), measured by the CCK‐8 assay. (*p < 0.05, **p < 0.01, and ***p < 0.001). Figure S2: The 1H NMR analysis of 1.5% n‐HA. Figure S3: (A) Frequency sweeps of 0.5% n‐HA, 0.8% n‐HA, and 1% n‐HA. The storage (G′) and loss (G′′) modulus were measured under a constant strain of 1% and angular frequency ranging from 0.1 to 100 rad/s at 25°C. (B) Viscosity of 0.5%, 0.8%, 1% n‐HA and ARTZ as a function of shear rate. (C) The difference in the yield stress of 0.5%, 0.8%, 1% n‐HA and ARTZ. (D) The variation of tan δ with frequency for 0.5%, 0.8%, 1% n‐HA and ARTZ. Figure S4: The degradation test of 0.5% n‐HA, 0.8% n‐HA, and 1% n‐HA under peroxidation (n = 3). [file BTM2-8-e10475-s001.docx]

# Supplemental Information

**Table S1 The specific primers used for different genes**

| **Gene** | **Sequence(5’-3’)** | |
| --- | --- | --- |
|  | **Forward** | **Reverse** |
| p16^INK4a^ | GGCCGATCCAGGTCATGATGATG | CACCAGCGTGTCCAGGAAGC |
| P21 | GATGGAACTTCGACTTTGTCAC | GTCCACATGGTCTTCCTCTG |
| Nrf2 | CAGCATAGAGCAGGACATGGAG | GAACAGCGGTAGTATCAGCCAG |
| COX-2 | GCGACATACTCAAGCAGGAGCA | AGTGGTAACCGCTCAGGTGTTG |
| SOD | TAACGCGCAGATCATGCAGCTG | AGGCTGAAGAGCGACCTGAGTT |
| IL-6 | TACCACTTCACAAGTCGGAGGC | CTGCAAGTGCATCATCGTTGTTC |
| IL-1β | TGGACCTTCCAGGATGAGGACA | GTTCATCTCGGAGCCTGTAGTG |
| IL-17 | CAGACTACCTCAACCGTTCCAC | TCCAGCTTTCCCTCCGCATTGA |
| COL2A1 | GATAACAGTCTTGCCCCACTTA | CAAGAACAGCATTGCCTATCTG |
| Aggrecan | GATCCTTACCGTAAAGCCCATC | CTCCAGTCTCATTCTCAACCTC |
| MMP3 | CTCTGGAACCTGAGACATCACC | AGGAGTCCTGAGAGATTTGCGC |
| MMP13 | CACTTTATGCTTCCTGATGACG | TCTGGCGTTTTTGGATGTTTAG |
| ADAMTS5 | GACCGATGGCACTGAATGTAGGC | TCTCCTCCACATACTCCGCACTTG |
| β-actin | GAAGATCAAGATCATTGCTCCT | TGGAAGGTGGACAGTGAG |

**Table 2 NMR data**

| Sample | (a) peak area | (m,n) peak area | MoD(%) |
| --- | --- | --- | --- |
| HA | 3.00 | 0 | 0 |
| 0.5% n-HA | 3.00 | 0.44 | 11 |
| 0.8% n-HA | 3.00 | 0.59 | 14.75 |
| 1.0% n-HA | 3.00 | 0.76 | 19 |
| 1.5% n-HA | 3.00 | 1.03 | 25.75 |

**
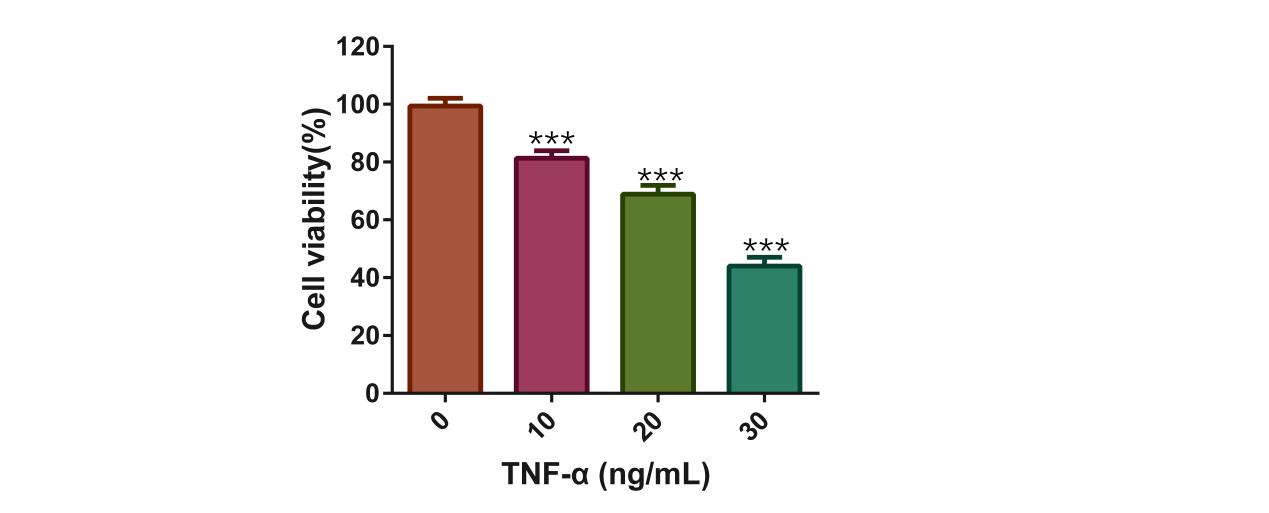
**

**Figure S1.** Establishment of TNF- α -induced inflammatory model. Cell viability of the chondrocytes following incubation with different concentrations of TNF-α (10, 20 and 30 ng/ml) , measured by the CCK-8 assay. (*p < 0.05, **p < 0.01, and ***p < 0.001).


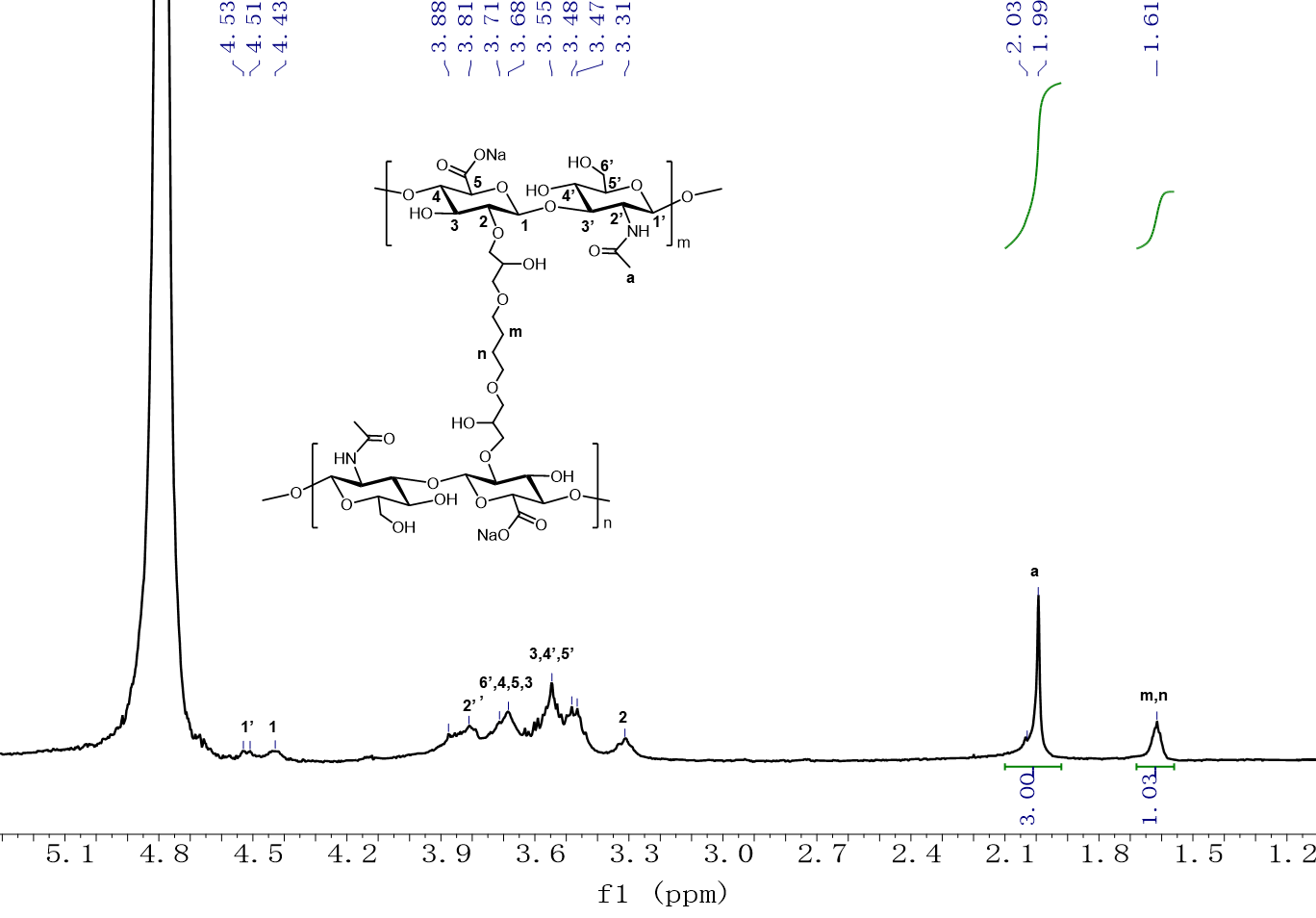


**Figure S2.** The ^1^H NMR analysis of 1.5% n-HA.


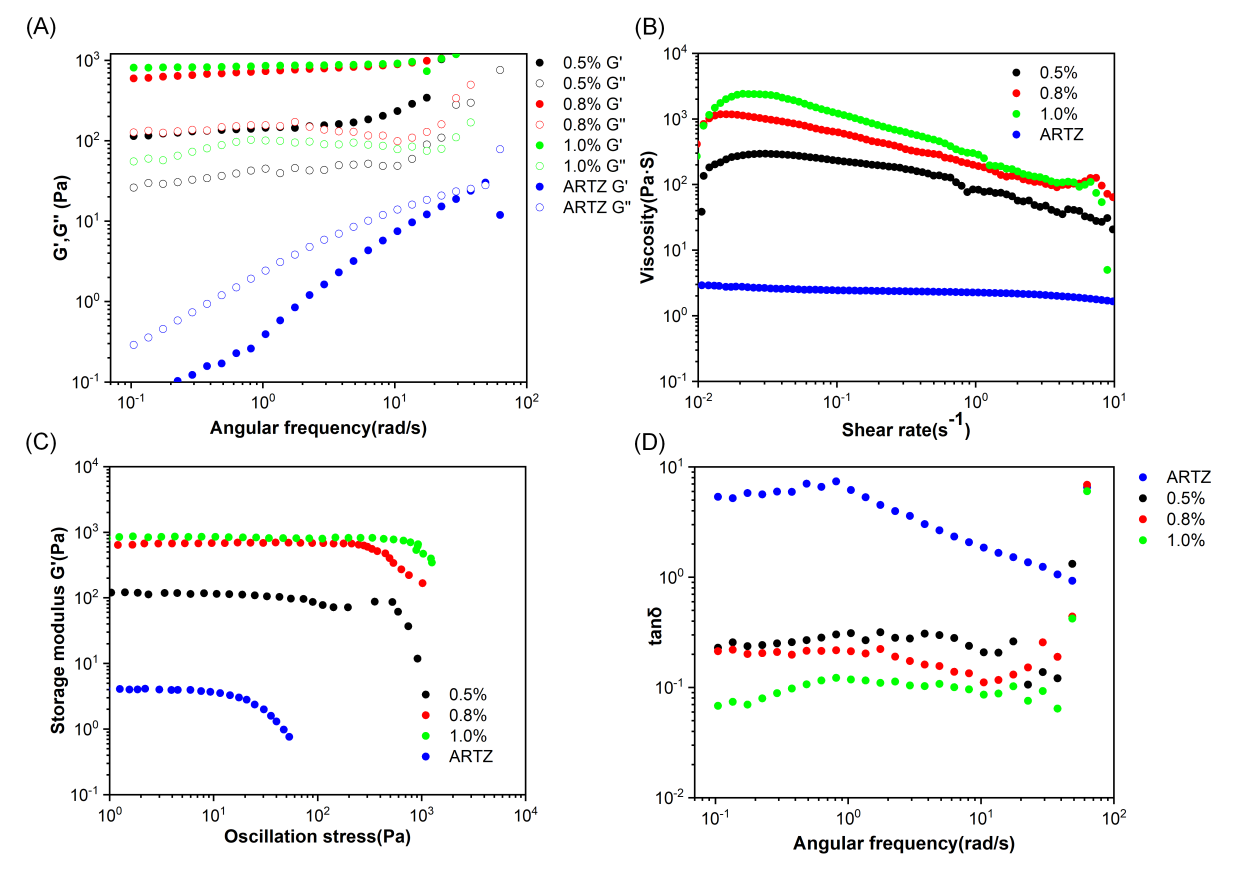


**Figure S3.** (A) Frequency sweeps of 0.5% n-HA, 0.8% n-HA and 1% n-HA. The storage (G’) and loss (G’’) modulus were measured under a constant strain of 1 % and angular frequency ranging from 0.1 to 100 rad/s at 25 °C. (B) Viscosity of 0.5%, 0.8%, 1% n-HA and ARTZ as a function of shear rate. (C) The difference in the yield stress of 0.5%, 0.8%, and 1% n-HA and ARTZ. (D) The variation of tan δ with frequency for 0.5%, 0.8%, 1% n-HA and ARTZ.

**
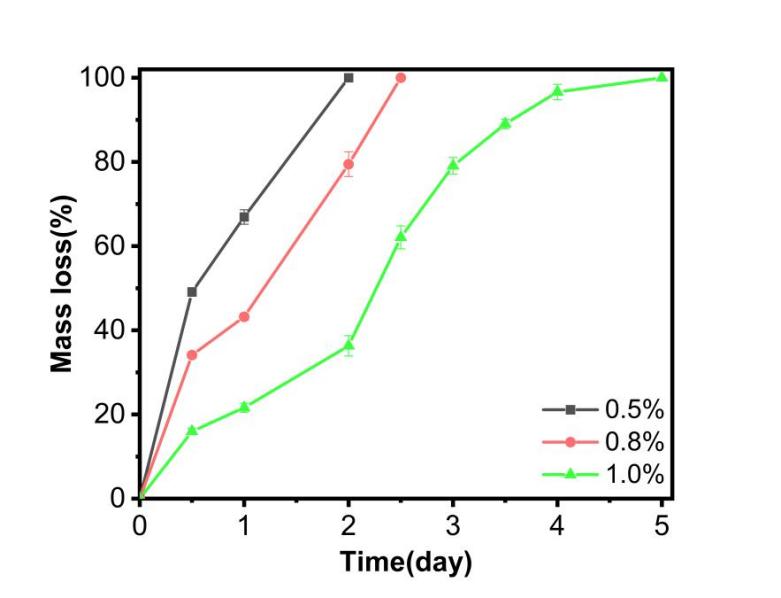
**

**Figure S4.** The degradation test of 0.5% n-HA, 0.8% n-HA and 1% n-HA under peroxidation (n=3).
